# The effectiveness and safety of *Kami Guibi-tang* for mild cognitive impairment: study protocol of a pilot, randomized, placebo-controlled, double-blind trial

**DOI:** 10.1186/s13063-019-3567-1

**Published:** 2019-07-22

**Authors:** Hee-Yeon Shin, Jeong-Hwa Kim, Geon-Ho Jahng, Woo-Sang Jung, Seong-Uk Park, Chang-Nam Ko, Jung-Mi Park

**Affiliations:** 1 Department of Clinical Korean Medicine, Graduate School, Kyung Hee University, 26, Kyungheedae-ro, Dongdaemun-gu, Seoul, 02447 Republic of Korea; 2 Department of Cardiology and Neurology, College of Korean Medicine, Kyung Hee University, 26, Kyungheedae-ro, Dongdaemun-gu, Seoul, 02447 Republic of Korea; 3 Department of Radiology, College of Medicine, Kyung Hee University Hospital at Gangdong, Kyung Hee University, #892, Dongnam-ro, Gangdong-gu, Seoul, 05278 Republic of Korea; 4 Stroke and Neurological Disorders Center, Kyung Hee University Hospital at Gangdong, 892, Dongnam-ro, Gangdong-gu, Seoul, 05278 Republic of Korea

**Keywords:** Mild cognitive impairment (MCI), *Kami Guibi-tang*, Herbal medicine, Seoul Neuropsychological Screening Battery (SNSB), Magnetic Resonance Imaging (MRI), Brain metabolite, Brain neurotransmitter, Cerebral blood flow (CBF)

## Abstract

**Background:**

Mild cognitive impairment (MCI) is an intermediate phase between normal aging and dementia. Since a majority of amnestic MCI (aMCI) cases progress to Alzheimer’s disease (AD), it is considered the prodromal stage of AD and, therefore, a treatment target for the prevention of further cognitive decline. However, there is no approved treatment for MCI at present. *Kami Guibi-tang* (KGT) is a herbal drug used in Korean medicine to treat amnesia, insomnia, loss of appetite, and depression. We will explore the effectiveness and safety of KGT in amnestic MCI in this trial.

**Methods/design:**

The study will be a single-center, randomized, placebo-controlled, double-blind trial. Eligible participants diagnosed with amnestic MCI will be randomly allocated to a treatment or control group. Participants will take KGT or placebo granules, three times a day, for 24 weeks. The primary outcomes will be changes in Seoul Neuropsychological Screening Battery (SNSB) scores, and magnetic resonance imaging (MRI) measurements including those of brain metabolites, neurotransmitters, and cerebral blood flow. The secondary outcomes will include the safety assessment, measured by changes in blood chemistry, changes in blood protein and cholesterol levels related to AD pathology, and a comparison of MRI changes between the two groups, using age and genotype as covariates.

**Discussion:**

This study will be the first clinical trial to identify the therapeutic potential of *Kami Guibi-tang* for amnestic MCI. The findings will provide insight into the feasibility of large-scale trials to gather evidence for KGT as a treatment for MCI.

**Trial registration:**

Korean Clinical Trial Registry, ID: KCT0002407. Registered on 30 March 2017.

**Electronic supplementary material:**

The online version of this article (10.1186/s13063-019-3567-1) contains supplementary material, which is available to authorized users.

## Background

Mild cognitive impairment (MCI) is an intermediate phase between normal aging and dementia. This clinical condition is characterized by self or caregiver-reported memory or cognitive complaints, and objective cognitive impairment, that is not severe enough to interfere with daily activities [1]. The majority of people with MCI develop dementia due to Alzheimer’s disease (AD) [2]. In particular, the amnestic subtype of MCI, which manifests as memory complaints, is often caused by degenerative etiologies and is generally regarded as a precursor of AD. A previous study revealed that 16% of amnestic MCI (aMCI) patients progress to dementia each year, 99% of whom receive an AD diagnosis [3]. Early treatment of mild-to-moderate AD is associated with better responses than later treatment, so treatment of MCI may delay progression to AD [4]. There is, however, no treatment approved for enhancing *memory function, or preventing further cognitive decline in MCI* [5]*.*

*Kami Guibi-tang* (KGT) is a popular herbal medicine used in Korean and Kampo medicine to treat amnesia, insomnia, loss of appetite, and depression. Several studies have explored the effects of KGT on cognitive impairment, particularly in AD. KGT improved learning performance in a mouse model of accelerated senescence [6], and reduced spatial memory impairment induced by scopolamine or delta-tetrahydrocannabinol [7]. In the 5XFAD mouse model of AD, KGT improved deficits in object recognition memory, and reversed the degeneration of cortical axons and presynaptic terminals [8]. A clinical study demonstrated that the Mini-Mental State Examination (MMSE) score of AD patients improved after receiving orally administered KGT extract granules for 3 months [9].

Previous studies have suggested that KGT could be beneficial for cognitive function in AD patients. However, few clinical trials have been published, and no study has yet investigated the effect of KGT on MCI. This pilot study aims to determine the effectiveness and safety of KGT for improving cognitive function, the influence on neuroimaging and neurochemical biomarkers, and the potential therapeutic effects in MCI.

## Methods/design

### Study design and setting

**Fig. 1:**
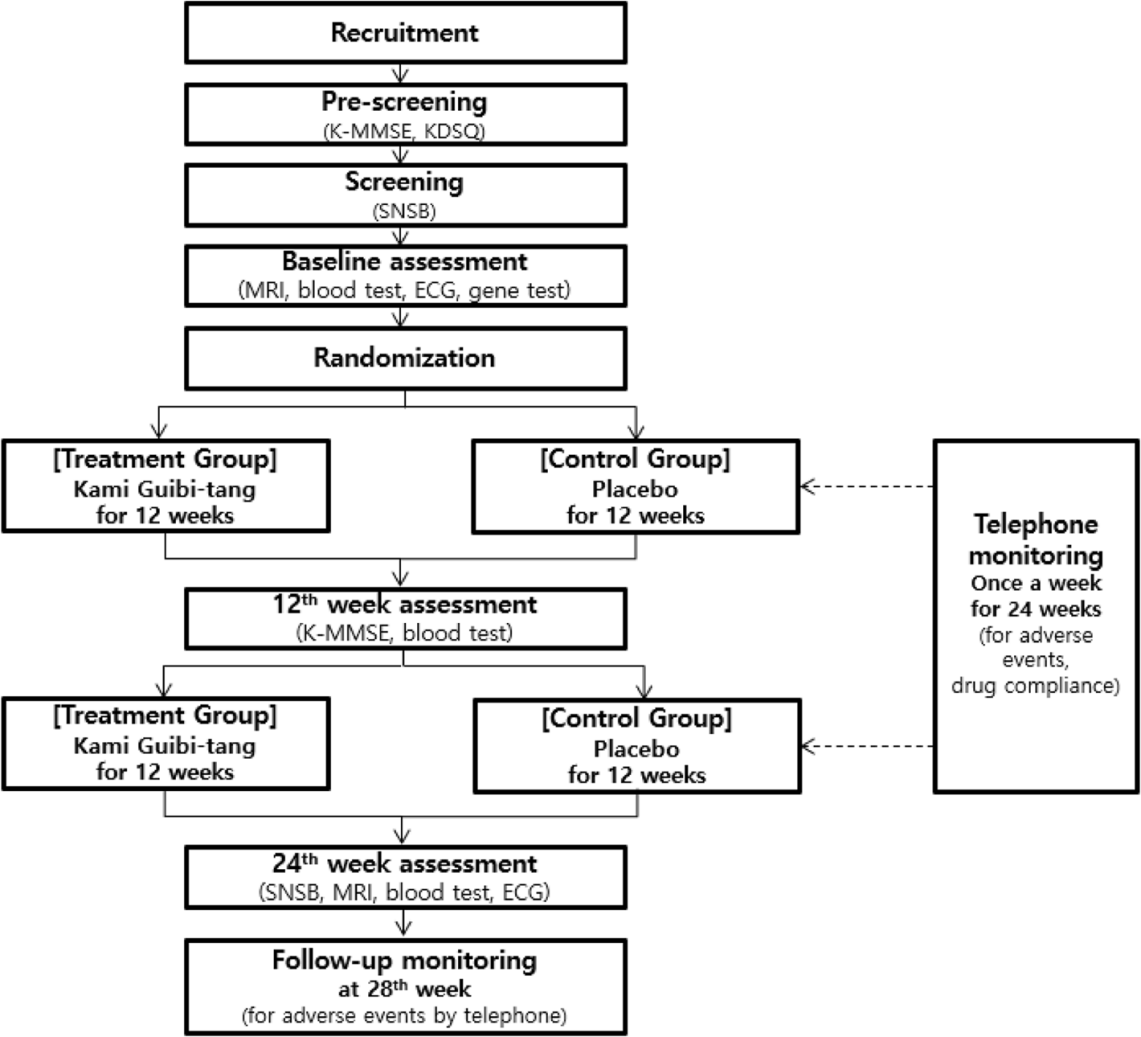
Flow chart of the study design

This study will use a single-center, randomized, double-blind, placebo-controlled, parallel-group clinical trial design. The study will take place at Kyung Hee University Hospital at Gangdong, Seoul, Korea from March 2017 through November 2019. The flow chart of the study design is shown in Fig. 1. The schedules for enrollment, interventions, and assessments are shown in Table 1.

**Table 1:**
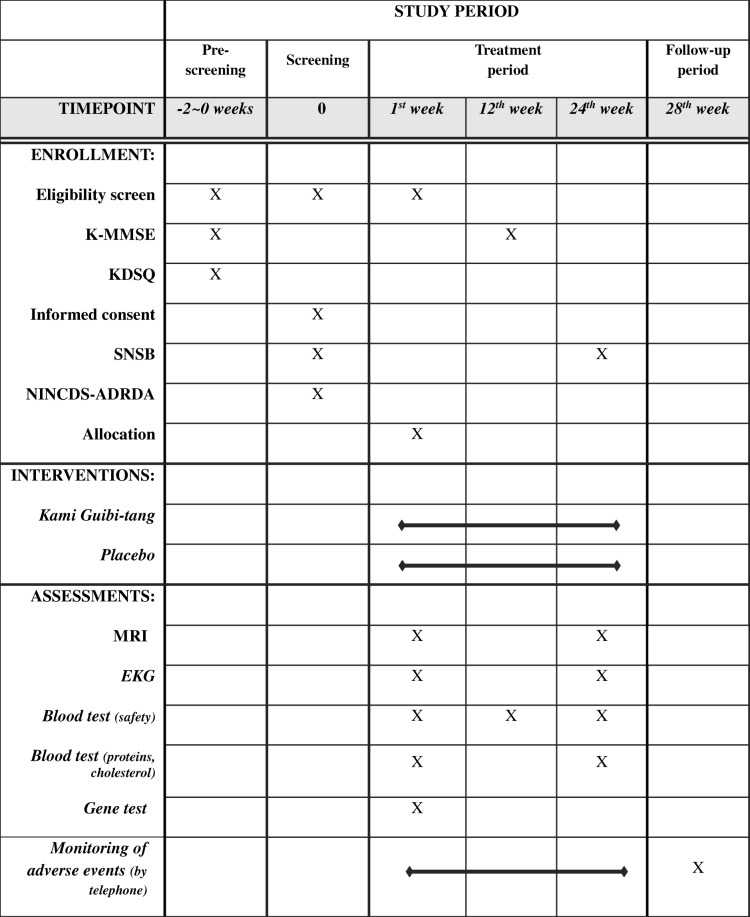
The schedule of enrollment, interventions, and assessments (SPIRIT 2013 Statement)

### Participants

#### Inclusion criteria

Participants who meet all of the following criteria will be eligible to participate:

1. Aged 55–90 years, with complaint of impaired memory
2. Objective cognitive impairment as measured by the Seoul Neuropsychological Screening Battery (SNSB), with a score of 3 on the Global Deterioration Scale (GDS), 0.5 on the Clinical Dementia Rating (CDR), and a normal score on the Korean MMSE (K-MMSE)
3. Diagnosed with aMCI by a neurologist
4. Participants who have not taken medication affecting cognitive function, including gliatilin, gliatamine, ginexin, tanamin, or other psychoactive drugs, in the previous 2 weeks
5. Participants who have not changed medication for underlying diseases in the previous 2 weeks, and no expected change in medication during the study period
6. No difficulty in communicating

#### Exclusion criteria

Participants who meet any of the following criteria will be ineligible to participate:

1. Diagnosed with AD, based on the criteria of National Institute of Neurological and Communicative Disorders and Stroke and Alzheimer’s Disease and Related Disorders Association (NINCDS-ADRDA)
2. Brain disorders causing neurological symptoms, other than cognitive impairment
3. Parkinson’s disease, Huntington’s disease, Down’s syndrome, Creutzfeldt-Jakob disease, or any other neurodegenerative disorder
4. Cognitive impairment resulting from other diseases including head trauma, hypoxic brain damage, vitamin deficiency, brain tumor, encephalitis, neurosyphilis, and mental retardation
5. Cerebrovascular diseases with magnetic resonance imaging (MRI) evidence
6. A previous or current history of major depression
7. Concomitant psychiatric disorders or behavioral problems that require antipsychotic medication
8. A history of a convulsive disorder, except for febrile convulsion during childhood
9. Unstable or life-threatening medical conditions
10. Uncontrolled hypertension
11. Heart or renal diseases
12. Peripheral edema
13. Gastrointestinal symptoms such as anorexia, nausea, abdominal pain, or diarrhea
14. Taking medication that could induce hypokalemia or myopathy
15. Drug hypersensitivity to the constituents of the study medication
16. Possibility of pregnancy
17. Clinically significant abnormalities in blood chemistry test results, including levels of serum aspartate aminotransferase/alanine aminotransferase (AST/ALT) more than two-fold the upper normal limit, or serum creatinine (Cr) level more than 10% of the upper normal limit
18. Participation in any other clinical trials within the previous 4 weeks
19. Illiteracy
20. Contraindications for MRI
21. Considered unsuitable for participation by the investigators

#### Drop-out criteria

1. Occurrence of any severe adverse effects
2. Participants’ voluntary withdrawal from the trial
3. Participants not following the protocol (i.e., drug compliance below 80%)
4. Use of additional medication to improve cognitive function during the study period
5. Decision made by the principal investigator

### Recruitment, enrollment

We will recruit a total of 38 patients through advertisements and referrals and screen the candidates aged 55–90 years, with complaints of impaired memory, using the inclusion/exclusion criteria, the K-MMSE, and the Korean Dementia Screening Questionnaire (KDSQ). Potentially eligible participants will undergo the SNSB, and those who are diagnosed with aMCI by a neurologist will be included in the trial. The investigators will provide a detailed explanation of the purpose, procedures, and potential risks and benefits of the study. Participants willing to participate in the trial and provide biological specimens will sign a consent form prior to enrollment.

### Randomization, allocation, blinding

A researcher uninvolved in the assessment will generate a random sequence using SPSS ver. 18. We will randomly allocate participants to either the treatment or the control group, in a 1:1 ratio using the block randomization method, with a block size of 4. The participants, assessor, clinical trial pharmacist, and researchers will be blinded to the allocations throughout the course of the study. Cases will be unblinded only if serious adverse events (SAE) occur.

### Intervention

After randomization, the treatment group will receive KGT granules (3 g/pack) and the control group will receive placebo granules (3 g/pack). *Kami Guibi-tang*, the herbal medicine under study, is composed of 14 drugs: *Astragali Radix* (1.0 g), *Ginseng Radix* (1.0 g), *Atractylodis Rhizoma* (1.0 g), *Poria cocos* (1.0 g), *Zizyphi Fructus* (0.67 g), *Zingiberis Rhizoma* (0.33 g), *Saussureae Radix* (0.33 g), *Glycyrrhizae Radix* (0.33 g), *Zizyphi Spinosi Semen* (1.0 g), *Longan Arillus* (1.0 g), *Angelicae Radix* (0.67 g), *Polygalae Radix* (0.67 g), *Bupleuri Radix* (1.0 g), *Moutan Radicis Cortex* (0.67 g), and *Gardeniae Fructus* (0.67 g). Treatment granules will be manufactured by Kyoung Bang Pharmaceutical Co., Ltd. (Incheon, Korea), which has been certified for Good Manufacturing Practice.

Placebo granules will be produced by the same manufacturer, using the standard method of placebo manufacturing according to the Korean Good Manufacturing Practice guidelines. The placebo will be similar to the *Kami Guibi-tang* granules in appearance, taste, and smell.

An independent clinical pharmacist will distribute KGT or placebo granules to the study participants. The pharmacist will instruct participants to dissolve the granules in hot water, stir well, and drink the solution three times per day, 30 min after meals, for 24 consecutive weeks. The researchers will call participants by phone once a week, reminding them to comply with the medication schedule.

The administration of medicine for underlying diseases, such as hypertension or diabetes mellitus, will be permitted during the intervention; however, any medication that can affect cognitive function will be prohibited. We will ask the participants to report all medications taken during the study at each visit, and record the names, duration, and dosage of the drugs on the case report form (CRF).

Participants will be required to return the unused trial drugs at the next visit. The number of returned drugs will be assessed to evaluate drug compliance, and participants with less than 80% compliance will be excluded.

### Outcome

Primary outcomes will include changes in cognitive function, and changes in imaging biomarkers, before and after taking KGT or placebo granules. For assessing cognitive function, the SNSB will be conducted and changes in SNSB results will be analyzed. For measuring the imaging biomarkers, brain MRI scans will be taken to assess the levels of brain metabolites, neurotransmitters, and cerebral blood flow (CBF).

Secondary outcomes will include the safety of KGT granules, and changes in blood-based biomarkers including proteins (amyloid β (Aβ) and tau) and cholesterols, and a comparison of MRI changes between the two groups using age and genotype as covariates. For safety assessments, blood chemistry tests for AST/ALT, glucose, blood urea nitrogen (BUN), Cr, Na, K, Cl, lactate dehydrogenase (LDH), creatine phosphokinase (CPK), and an electrocardiogram (ECG) will be conducted. Blood biomarkers including Aβ protein, tau protein, high-mobility group box (HMGB), small EDRK-rich factor (SERF) 1A, and cholesterol derivatives will also be measured. *APOE* genotype testing will be performed. Detailed explanations are shown below.

### Assessments

#### K-MMSE and SNSB

The K-MMSE will be performed at the screening visit and after 12 weeks of medication, to screen for MCI and assess the cognitive status of participants. This is a simple screening test for the longitudinal assessment of general cognition [10].

The full version of the SNSB-II will be measured at baseline and after 24 weeks of medication, to evaluate the effect of *Kami Guibi-tang* on cognitive function. The change in the mean of the SNSB scores, including the SNSB for dementia (SNSB-D) scores, will be compared between the KGT and placebo groups as a primary outcome.

The SNSB is one of the standardized neuropsychological test batteries, widely used in South Korea, for assessing general cognitive abilities. The test is composed of multiple subtests, which evaluate five cognitive aspects: attention, memory, language, visuospatial function, and executive function. Other related tests are included, such as the K-MMSE, Clinical Dementia Rating (CDR), Barthel–Activities of Daily Living (BADL), and Geriatric Depression Scale (GDS). The estimated completion time of the whole battery is 1.5–2 h [11].

A modified version of the original SNSB for dementia (SNSB-D) will be used to provide a general cognitive functioning (GCF) score, drawn from the sum of five domains. The maximum total score is 300 points consisting of 17/300 (6%) for attention, 27/300 (9%) for language and related function, 36/300 (12%) for visuospatial function, 150/300 (50%) for memory, and 70/300 (23%) for frontal/executive function [12]. The contents of the SNSB-D are shown in Table 2.

**Table 2:** Contents of the Seoul Neuropsychological Battery for Dementia (SNSB-D)

Domains (total score)	Subtests	Maximum points
Attention (17)	Digit span forward	9
	Digit span backward	8
Language and related function (27)	K-BNT	15
	Calculation	12
Visuospatial function (36)	RCFT copy	36
Memory (150)	Orientation	6
	SVLT free/delayed recall	48
	SVLT recognition	12
	RCFT free/delayed recall	72
	RCFT recognition	12
Frontal/executive function (70)	Motor impersistence	3
	Contrasting program	3
	Go-no-go test	3
	Fist-edge-palm	3
	Luria loop	3
	Categoric word generation	20
	Phonemic word generation	15
	Stroop test-color reading	20
GCF score		300

*K-BNT* Korean short version of the Boston Naming Test, *RCFT* Rey Complex Figure Test, *SVLT* Seoul Verbal Learning Test, *GCF* global cognitive function

#### MRI

Participants will undergo brain MRI at baseline and after 24 weeks of medication, to measure changes in brain metabolite and neurotransmitter levels, cerebral blood flow, tissue volume, and to evaluate brain abnormalities.

Proton magnetic resonance spectroscopy (^1^H-MRS) will be used to measure brain metabolite and neurotransmitter levels. Single-voxel Point-RESolved Spectroscopy (PRESS) MRS will be performed at the precuneus and posterior cingulate area of the brain, with a voxel size of 30 mm × 30 mm × 30 mm, to detect *N*-acetylaspartate (NAA) and glutamate-glutamine complex (Glx, with both Glu and Gln). *N*-acetylaspartate and Glx will be quantified using LCModel software. MEshcher-GArwood (MEGA) PRESS MRS will be performed to detect gamma-aminobutyric acid (GABA) in the same area as the PRESS MRS. The amount of GABA will be quantified using GANNET software. Pseudo-continuous arterial spin-labeling (pCASL) MRI will be performed to measure cerebral blood flow (CBF) in the brain. Voxel-based CBF will be mapped using local software. A three-dimensional T1-weighted image will be acquired using the magnetization-prepared rapid gradient-echo (MPRAGE) sequence, to quantify the gray and white matter tissue volume in the brain. T2-weighted turbo-spin echo (TSE), and fluid attenuation inversion recovery (FLAIR) sequences, will be used to evaluate brain abnormalities in participants. Changes in CBF and mean levels of NAA/Cr, Glx/Cr, and GABA/Cr, will be compared between KGT and placebo groups, as a primary outcome.

#### Blood testing

Amyloid β protein, tau protein, HMGB, SERF 1A, and cholesterol derivatives will be measured in the blood at baseline and after 24 weeks of medication to observe changes in serum proteins and cholesterol associated with AD pathology. Changes in mean values of blood biomarker levels will be compared between the KGT and placebo groups.

#### Genotyping

Apolipoprotein E (*APOE*) epsilon genotyping (e23, e33, e34, e44) will be performed using blood samples at baseline, to determine if the effect of KGT is influenced by *APOE* genotype. The changes in mean values of NAA/Cr, Glx/Cr, GABA/Cr, and CBF before and after the intervention will be compared between carriers and non-carriers of the *APOE* epsilon 4 allele.

#### Safety assessment

Laboratory tests and ECGs will be performed for safety outcomes. Blood levels of AST/ALT, glucose, BUN, Cr, Na, K, Cl, cholesterol, LDH, and CPK will be measured at baseline, 12, and 24 weeks after medication. An ECG will be performed at baseline, and 24 weeks after medication. Vital signs will be recorded at every visit. Any abnormal results in laboratory tests, ECG or vital signs, will be closely monitored. The investigators will call participants by phone 4 weeks after completion of medication, to monitor occurrence of any adverse events.

### Data management and monitoring

Case report forms (CRFs) will be used for each participant to collect relevant data. To promote data quality and accuracy, one trained investigator will complete the CRFs and a second investigator will independently review all CRFs. All documents will be kept at the study site, and the data will be entered and stored in a password-protected computer. All procedures will comply with confidentiality standards for medical data. All documents and collected data will be kept for 3 years after completion of the study, and will then be destroyed. The data management process will be monitored by an independent agent. Only the investigators and the monitoring agent will have access to the dataset. Auditing will be conducted by the Korean Ministry of Food and Drug Safety.

### Statistics

#### Sample size calculation

This clinical trial is a pilot study to examine the feasibility of a full randomized clinical trial of KGT, and to determine the sample magnitude required for large-scale studies. To our knowledge, no prior study has investigated the effect of KGT on MCI. There are no previous data indicating the sample size needed to yield statistically significant results for determining the effect of KGT by the SNSB scores. For pilot trials, the sample size of 10–20 participants per group was suggested by Kieser and Wassmer. [13]. Browne recommended using at least 30 subjects to estimate parameters [14]. Our target sample size is 30, so 38 individuals will be recruited to allow for a 20% dropout rate.

#### Data analyses

The investigators, along with an independent professional statistician, will perform the data analyses. All statistical analyses will be performed using SPSS for Windows. The effectiveness test will be performed using the intention-to-treat (ITT) principle and the per-protocol (PP) principle. Missing data will be adjusted with the last-observation-carried-forward (LOCF) imputation method. The safety test will be analyzed using the ITT principle, without adjustment. We will not perform interim analysis.

The student’s *t* test will be used for parametric variable comparisons between the two groups, and the paired *t* test will be used for intra-group comparisons. The Mann-Whitney *U* test will be used for non-parametric variable comparisons between the two groups, and the Wilcoxon signed-rank test will be used for intra-group comparisons.

The paired *t* test will be used to compare changes in MRI measurements, blood proteins, and cholesterol levels, before and after treatment. Correlation analysis will be used to estimate the relationship between the changes in MRI measures and changes in blood test results. Linear regression analysis will be used to compare changes in MRI measures, between the study group and the placebo group, using age and *APOE* genotype as covariates. Statistical significance will be set at *P* < 0.05, and all tests will be two-tailed.

### Adverse events

An undesirable, unexpected sign, symptom, or disease that occurs during the trial will be identified as an adverse event (AE), regardless of any causal relationship with the study intervention. Adverse events will be checked for at every visit, from assessment of participants’ subjective reports and by objective examination, including blood tests and ECGs. Monitoring will also be performed by phone each week during the intervention period, and 4 weeks after the end of treatment. All AEs will be recorded in the CRF by the site investigator and assessed for severity and causality. Details of each AE will be recorded, including start and end date, feature, duration, severity, and causal relationship to the study medication.

Serious adverse events (SAEs) are defined as illness requiring hospitalization, events that result in persistent or significant disability or incapacity, events deemed life-threatening, death, a congenital anomaly or birth defect, or other important medical conditions. If SAEs occur, study participation will be discontinued, appropriate measures will be taken immediately, and the Institutional Review Board (IRB) will be notified as soon as possible. All occurrences of AEs will be monitored until they subside or stabilize.

## Discussion

Alzheimer’s disease has become one of the most serious health concerns, due to increasing life span and aging populations worldwide. The disease is a chronic, progressive neurodegenerative disorder, characterized by pathological changes including neuritic plaques and neurofibrillary tangles, which accumulate years before clinical symptoms manifest [15]. Early treatment of MCI patients could halt or slow down irreversible progression of neurodegeneration to AD [16]. Nevertheless, there is no proven treatment for MCI to date [17]. In this trial, we will explore the potential of KGT as a therapeutic agent for MCI patients.

Clinical symptoms are not evident in MCI, so we will utilize an extensive assessment to evaluate the effect of KGT on cognitive function and disease progression. Instruments, such as the K-MMSE and the Alzheimer’s Disease Assessment Scale-cognitive subscale (ADAS-cog), have been mainly used in previous studies for measuring cognitive decline. However, they lack sensitivity in detecting mild degrees of cognitive dysfunction, which demonstrate ceiling effects in MCI [18]. We will use the full neuropsychological battery, the SNSB, which contains comprehensive and diverse tests with varying difficulties, to sensitively and accurately monitor changes in cognitive function [11].

We will also measure neuroimaging markers that reflect dynamic neuropathological processes. Structural MRI-based measurements of brain atrophy are the most widely accepted marker of AD progression [19]. However, the utility of structural MRI for MCI is limited because overt loss of neurons and associated brain atrophy occur in the later stages of AD [20]. Functional changes also occur in the brains of MCI and AD patients. There is consistent reduction of cerebral metabolism, blood flow, and disturbances in neurotransmission that precede substantial brain atrophy [21]. Therefore, changes in brain metabolite and neurotransmitter levels, and cerebral blood flow will be measured in this trial.

Proton MRS is a sensitive method for estimating brain metabolite and neurotransmitter concentrations [22], including NAA, myo-Inositol (mI), choline (Cho), Glx, and GABA, frequently measured as ratios to creatine [23]. A decrease in concentrations of NAA, a neuronal metabolite, and Glx, a component of the excitatory neurotransmitter system in the brain, is indicative of neuronal dysfunction in AD [24]. Detecting changes in neuronal markers may be suitable for assessing treatment responses.

Arterial spin-labeling perfusion MRI (ASL MRI) is used to quantify regional cerebral blood flow (rCBF) [25]. Alzheimer’s disease patients show regional hypoperfusion that reflects patterns of reduced brain functional activity, using this technique [26]. Studies have suggested that ASL MRI is appropriate for examining neural response to pharmacological agents [27]. Prior studies that measured rCBF used Single Photon Emission Computed Tomography (SPECT). However, ASL MRI is advantageous because there is no exposure to ionizing radiation or the need for intravenously administered contrast agents, and there is higher accessibility, lower cost, and higher spatial resolution [26].

As secondary outcomes, we will observe changes in blood-based biomarkers levels, including Aβ protein, tau protein, HMGB, SERF 1A, and cholesterol derivatives, which are proposed to be associated with AD pathology [28]. Aβ protein and tau proteins are the main feature of AD neuropathology, and are closely associated with neuronal loss. High plasma Aβ levels are a risk factor for AD [29]. High total cholesterol levels accelerate the production of Aβ protein in AD, and are related to increased cognitive impairment in humans [30]. The strongest genetic risk factor for AD, *APOE* genotype, will also be determined by plasma *APOE* proteins [31]. This genetic marker will provide information for distinguishing which patients are most likely to benefit from KGT administration.

This pilot study will be the first rigorous clinical analysis of KGT for the treatment of aMCI, and should provide evidence for the effectiveness and safety and of KGT. Our extensive assessment, using various biomarkers, should reveal the mechanisms underlying the effect of KGT on disease progression. The findings will support a large-scale confirmatory clinical trial to gather evidence for KGT use in MCI patients.

There are some limitations in this protocol. The target sample size is relatively small, and the sample size could not be calculated by standard methods. However, this is a pilot study to generate data for sample size calculations in a future randomized trial, which will include a larger sample size. Also, the total intervention period is 24 weeks and the monitoring period after the completion of taking medicine is 4 weeks, which is relatively short considering the nature of the disease. Long-term follow-up will be necessary in future trials.

## Trial status

This study began recruitment in May 2017 and we have currently enrolled 33 participants.

## Additional file

Additional file 1: Standard Protocol Items: Recommendations for Interventional Trials (SPIRIT) Checklist. (DOCX 42 kb)

## Data Availability

Not applicable.

## Abbreviations

AD: Alzheimer’s disease
ADAS-cog: Alzheimer’s Disease Assessment Scale-cognitive subscale
ALT: Alanine aminotransferase
aMCI: Amnestic mild cognitive impairment
ASL MRI: Arterial spin-labeling perfusion magnetic resonance imaging
AST: Aspartate aminotransferase
BUN: Blood urea nitrogen
CBF: Cerebral blood flow
CDR: Clinical Dementia Rating
GDS: Global Deterioration Scale
KDSQ: Korean Dementia Screening Questionnaire
KGT: *Kami Guibi-tang*
K-MMSE: Korean Version of Mini-Mental State Examination
MCI: Mild cognitive impairment
MRS: Magnetic resonance spectroscopy
NINCDS-ADRDA: National Institute of Neurological and Communicative Disorders and Stroke and Alzheimer’s Disease and Related Disorders Association
pCASL MRI: Pseudo-continuous arterial spin-labeling perfusion magnetic resonance imaging
rCBF: Regional cerebral blood flow
SNSB: Seoul Neuropsychological Screening Battery
